# Physiological phenotyping of mammalian cell lines by enzymatic activity fingerprinting of key carbohydrate metabolic enzymes: a pilot and feasibility study

**DOI:** 10.1186/s13104-019-4697-y

**Published:** 2019-10-22

**Authors:** Julian Geiger, Rebecca Doelker, Sofia Salö, Thomas Roitsch, Louise T. Dalgaard

**Affiliations:** 1 Department of Science and Environment, Roskilde University, Universitetsvej 1, 4000 Roskilde, Denmark; 2 Section for Crop Sciences, Department of Plant and Environmental Sciences, Copenhagen Plant Science Centre, University of Copenhagen, Højbakkegårds Allé 13, 2630 Taastrup, Denmark; 3 Global Change Research Institute CAS, Drásov, Czech Republic

**Keywords:** INS-1E, Enzyme assays, 96 well format, Aldolase, Hexokinase, Glucose-6-phosphate dehydrogenase, Phosphoglucoisomerase, Phosphoglucomutase, Phosphofructokinase

## Abstract

**Objective:**

Enzymatic fingerprinting of key enzymes of glucose metabolism is a valuable analysis tool in cell physiological phenotyping of plant samples. Yet, a similar approach for mammalian cell line samples is missing. In this study, we applied semi-high throughput enzyme activity assays that were originally designed for plant samples and tested their feasibility in extracts of six frequently used mammalian cell lines (Caco2, HaCaT, C2C12, HEK293, HepG2 and INS-1E).

**Results:**

Enzyme activities for aldolase, hexokinase, glucose-6-phosphate dehydrogenase, phosphoglucoisomerase, phosphoglucomutase, phosphofructokinase could be detected in samples of one or more mammalian cell lines. We characterized effects of sample dilution, assay temperature and repeated freeze–thaw cycles causing potential biases. After careful selection of experimental parameters, the presented semi-high throughput methods could be established as useful tool for physiological phenotyping of cultured mammalian cells.

## Introduction

Deciphering metabolic events based solely on mRNA expression or protein data potentially draws an incomplete or even misleading picture. Transcripts and protein abundance often poorly correlate. The correlations are typically worse, when it comes to comparisons between transcript levels and enzyme activities that are controlled by various additional determinants, such as activating or inhibiting factors. Thus, additional data on enzyme activities can provide valuable, additional information. To this end Jammer et al. [1] developed a series of spectrophotometric assays adapted for a 96 well microplate format to measure activities of key enzymes in plant carbohydrate metabolism. Despite potential benefits, a similar approach has not been developed for samples of mammalian cells. To overcome this limitation, we here piloted the application of the original method for physiological phenotyping of plants [2] to determine an enzyme activity signature in mammalian cells [1]. We used modified protocol steps for harvesting and lysis suitable for cultured cells and tested six widely used mammalian cell lines: Caco2, HaCaT, C2C12, HEK293, HepG2 and INS-1E. The clonal insulin secreting β-cell line INS-1E was utilized to perform extensive testing on four of the six enzymes [3, 9]. The other five mammalian cell lines constitute models of cell types important for whole body metabolism: Caco2 (intestinal cells) [4], HaCaT (skin keratinocytes) [5], C2C12 (myoblasts) [6], HEK293 cells (undifferentiated cells from embryonic kidney) [7] and HepG2 (hepatocytes) [8]. The cell lines used were selected to represent a variety of widely used models for differentiated tissues, thus demonstrating the potential of the original method to be applied to biomedical experimental research models.

## Main text

### Methods

#### Cell culture

Caco2, a human intestinal epithelial cell line [4], was cultured in DMEM with 20 mM glucose, P/S and 10% fetal bovine serum (FBS) [10]. HaCaT [5], HEK293 [7], C2C12 [6] and HepG2 cells [8] were cultured in DMEM with 25 mM glucose, 10% FBS and P/S. ⁠INS-1E cells (a kind gift of Claes Wollheim [3, 9]) were cultured in RPMI 1640 with Glutamax supplemented with 100 U/mL penicillin and 100 µg/mL streptomycin (P/S), 10% heat-inactivated FBS, and 50 µM β-mercaptoethanol as previously described [11].

To produce enzyme lysates, cells were seeded in T25 flasks (Falcon). The number of cells seeded depended on cell line: Caco2: 2 × 10^5^, HaCaT: 2.5 × 10^5^, C2C12: 8 × 10^4^, HEK293: 2 × 10^5^, HepG2: 2.5 × 10^5^, INS-1E: 1 × 10^6^. This amount of cells gave ample material for the presented experiments and smaller growth vessels would often suffice. Cells were harvested 72 h after seeding at 80–90% confluence.

#### Generation of cellular extracts

Cells were washed with PBS and harvested by addition of 0.05% trypsin–EDTA. After addition of medium, trypsin was removed by centrifugation at 200×*g*, 5 min, 4 °C. Cells were washed in PBS, centrifuged at 200×*g*, 5 min, 4 °C and resuspended in 1 mL PBS supplemented with 0.1% Triton X-100 and kept on ice. After sonication in a 1.5 mL microcentrifuge tube on ice for 2 × 10 s, 100 Hz (Vibra-cell, Sonics & Material), cells were centrifuged in a pre-cooled table centrifuge (AccuSpin, Fisher Scientific) at 12,000×*g*, 4 °C, for 30 min. The supernatant was split into 200 µL aliquots and snap-frozen in liquid nitrogen for storage at − 80 °C. Protein concentration of cellular lysates was determined using a BCA-assay kit (Pierce) following the microplate protocol.

#### Enzyme activity assays

Enzyme activity assays for aldolase (Aldo), hexokinase (low Km) (HK), glucose-6-phosphate dehydrogenase (G6PD), phosphoglucomutase (PGM), phosphoglucoisomerase (PGI) and phosphofructokinase (PFK) were performed as described previously [1] (Additional file 1). In short, 10 µL of cellular lysates were mixed with assay buffers, substrates and, if applicable, auxiliary enzymes in 96-well plates (160 µL reaction volume). NAD(P)H absorbance was monitored for 40 m at 340 nm and 30 °C (unless otherwise noted) in a ELISA plate reader (BioTek Elx808 or Synergy 2) using UV-transmissive flat bottom 96-well plates (UV-Star; Greiner Bio One). Data were analyzed by linear regression of the linear absorbance curve using Gen5 (V 2.05) plate reader software and presented as specific activities. Additional file 1 contains a detailed excel sheet template describing the protocol for performing the enzyme activity assays.

#### Data and statistical analysis

All samples were assayed in three technical replicates with one substrate negative control on the same plate using the same reaction mix to ensure robustness and reproducibility. Protein concentrations were used for normalization of enzyme activities. Normalization to cell counts per sample yielded similar results (data not shown). Data analysis and statistical evaluation was done in Excel and Graph Pad Prism, respectively. Data were normally distributed. Differences between samples were evaluated by Student’s t-test, paired t-test or one-way ANOVA (repetitive measures) as appropriate and p < 0.05 was considered significant.

### Results

#### Activities of glucose metabolic enzymes in mammalian cell lines

As examples of tissues important for whole body glucose metabolism, six commonly used mammalian cell lines were chosen for the study; Caco2, HaCaT, C2C12, HEK293, HepG2, and INS-1E, to represent in vitro models of intestinal enterocytes, skin keratinocytes, skeletal muscle myoblasts, undifferentiated embryonic cell type, liver hepatocytes and pancreatic β-cells, respectively.

**Fig. 1:**
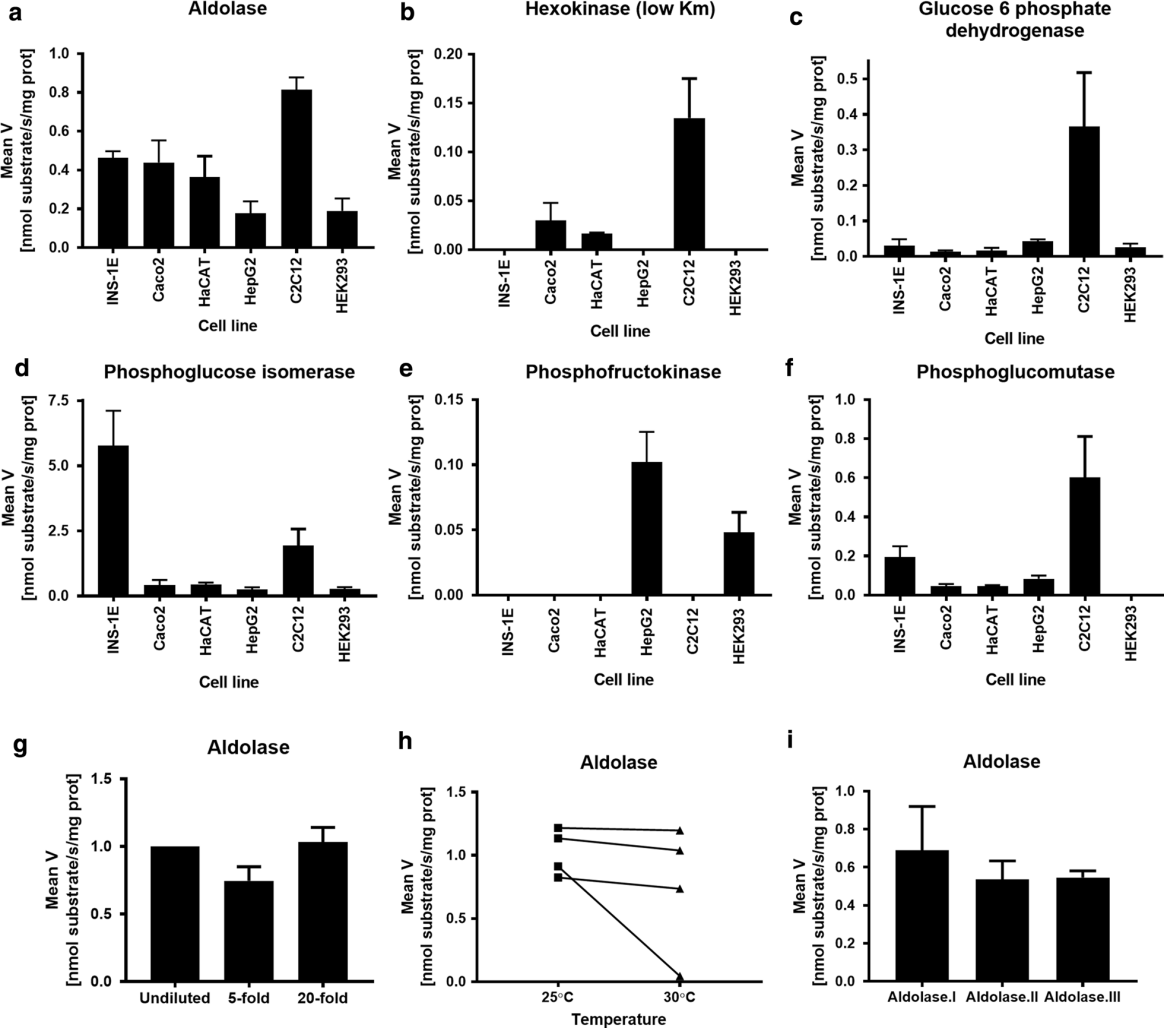
Activities of glucose metabolic enzymes in Caco2, HaCaT, C2C12, HEK-293, HepG2, and INS-1E cells. **a** Aldolase (Aldo), **b** Hexokinase (low Km) (HK), **c** Glucose 6 phosphate dehydrogenase (G6PD), **d** Phosphoglucose isomerase (PGI), **e** Phosphofructokinase (PFK) and **f** Phosphoglucomutase (PGM). N = 4–6 extracts tested per cell line. Data are shown as mean ± SEM. Testing of parameters on aldolase activity assays in INS-1E samples: **g** Dilution series of cell extracts (N = 3). Diluted samples were normalized to the level of the undiluted sample (set to 1). **h** Temperature sensitivity was tested by assay performance at 25 °C and 30 °C. N = 4 cell extracts tested per temperature. **i** Sensitivity to freeze–thaw cycles was assessed using cell extracts that underwent 1 (I), 2 (II) or 3 (III) freeze-thaw cycles. N = 4 cell extracts tested per condition. Data are shown as mean ± SEM. Additional data relating to this figure can be found in Additional file 2

We generated crude extracts and performed activity assays for Aldo, HK, G6PD, PGM, PGI, and PFK (Fig. 1). All six enzyme activities were detectable in one or more cell line (Fig. 1). The observed activities reflected the tissue-of-origin of the cell lines: The highly proliferating and glycolytic myoblast cell line, C2C12 showed high activity of all enzymes except for PFK. PFK was determinable at high activity only in the liver derived HepG2 cells (Fig. 1e). Aldo and PGI had high activity in all tested cell lines (Fig. 1a, d), whereas the HK (low Km) activity was only observed in Caco2, HaCaT and C2C12 cells (Fig. 1b). INS-1E cells express mainly glucokinase, the high Km hexokinase [12], and therefore does not display discernable activity. Increasing the assay glucose concentration to 15 mM led to an average activity of 1.4 nmol/s/mg protein lysate in INS-1E cells. G6PD activity (Fig. 1c) was observed at low levels in INS-1E, Caco2, HaCaT and HepG2 cells, while C2C12 cells had high activity, indicating cell line specific use of the pentose phosphate pathway. PGM activity, indicating glucose flux towards glycogen metabolism, was easily detectable in all cell lines, except HEK293 (Fig. 1f).

#### Robustness of enzyme assays showing activity in INS-1E cells: aldolase, glucose-6-phosphate dehydrogenase, phosphoglucose isomerase, and phosphoglucomutase

Since INS-1E β-cells displayed high activity in the aldolase, G6PD, PGI and PGM assays, we used this cell line to further assess assay precision (Table 1), lysate dilution (Fig. 1g), sensitivity to temperature (Fig. 1h) and freeze–thaw cycles (Fig. 1i). Assay precision was estimated from 3 to 4 measurements of one sample of INS-1E cell extract (Table 1) and ranged from an intra assay CV of 7.2% for G6PD to 15.7% for PGI. This is an acceptable degree of variation for replicate measurements, although it could be beneficial to increase number of replicate measurements of PGI activity. Day-to-day variation in raw data was large for these assays (18-106%) and it is thus necessary to include inter assay calibrators or standards, if the amount of samples exceeds the capacity to measure in 1 day. Of note, the relative difference between samples measured at different days, was low (data not shown). We determined the variation of the combined experimental procedure: cell culture, harvest, lysate preparation, protein concentration and enzyme activity assay by assaying 9–12 different INS-1E cell extracts derived from independent samples from 3 different passages: the CV between samples ranged from 14.6 to 16.7% for the 4 enzyme activity assays when measured in the same enzyme assay setup (Table 1), which is acceptable for most basic laboratory analyses.

**Table 1:** Enzyme assay performances

Enzyme assay performance				
Enzyme	Mean V (nmol/s/mg prot)	Standard deviation	N	Intra assay CV (%)
Aldolase	1.07	0.08	4	7.7
G6PD	0.16	0.01	4	7.2
PGI	12.0	1.9	3	15.7
PGM	0.81	0.07	3	9.2

Performance of entire experimental procedure				
Enzyme	Mean V (nmol/s/mg prot)	Standard deviation	N	Sample CV (%)
Aldolase	1.07	0.16	12	16.1
G6PD	0.16	0.06	11	14.6
PGI	12.0	2.0	9	16.4
PGM	0.84	0.12	9	14.8

*Aldo* Average enzymatic activities of aldolase, *G6PD* lucose 6 phosphate dehydrogenase, *PGI* phosphoglucose isomerase and *PGM* phosphoglucomutase determined in INS-1E cell lysate, and average enzymatic activities of Aldo, G6PD, PGI and PGM determined in biological replicates of INS-1E cell extracts derived from 3 different cell passages

To investigate the sensitivity of the Aldo, G6PD, PGI and PGM activity assays to sample amount we tested 5 µL undiluted cell extracts versus 5-fold and 20-fold dilutions (Fig. 1g, Additional file 2: Figure S1a–c). Aldo and PGM activities were largely unaffected by dilution, whereas the G6PD assay could not detect 20-fold diluted cell lysates. Consequently, fivefold dilution of cell extract is possible for all four assays, although this would depend on the cell line assayed.

The assays’ temperature dependency was tested at 25 °C and 30 °C using the same set of four samples. While Aldo and G6PD activities did not change significantly between 25 °C and 30 °C, PGI activity decreased 26% at 30 °C (P < 0.0001) and PGM activity increased 82% at 30 °C (P < 0.05) (Fig. 1h, Additional file 2: Figure S1d–f). Thus, while enzyme activity for PGI and PGM assays are within measurable ranges at both 25 °C and 30 °C, these assays are markedly influenced by assay temperature.

Enzymes often show sensitivity towards freeze–thaw cycles. We therefore tested Aldo, G6PD, PGI and PGM activities in cell extracts thawed once, twice or thrice. Only Aldo activity was preserved after freeze–thawing three times (Fig. 1i), while G6PD, PGI and PGM activities decreased markedly (Additional file 2: Figure S1g–i). Thus, it is advisable to aliquot extracts and minimize the number of freeze–thaw cycles.

#### Case study: palmitate-induced alterations in enzyme gene expression and activity follow dissimilar trends in INS-1E β-cells

**Fig. 2:**
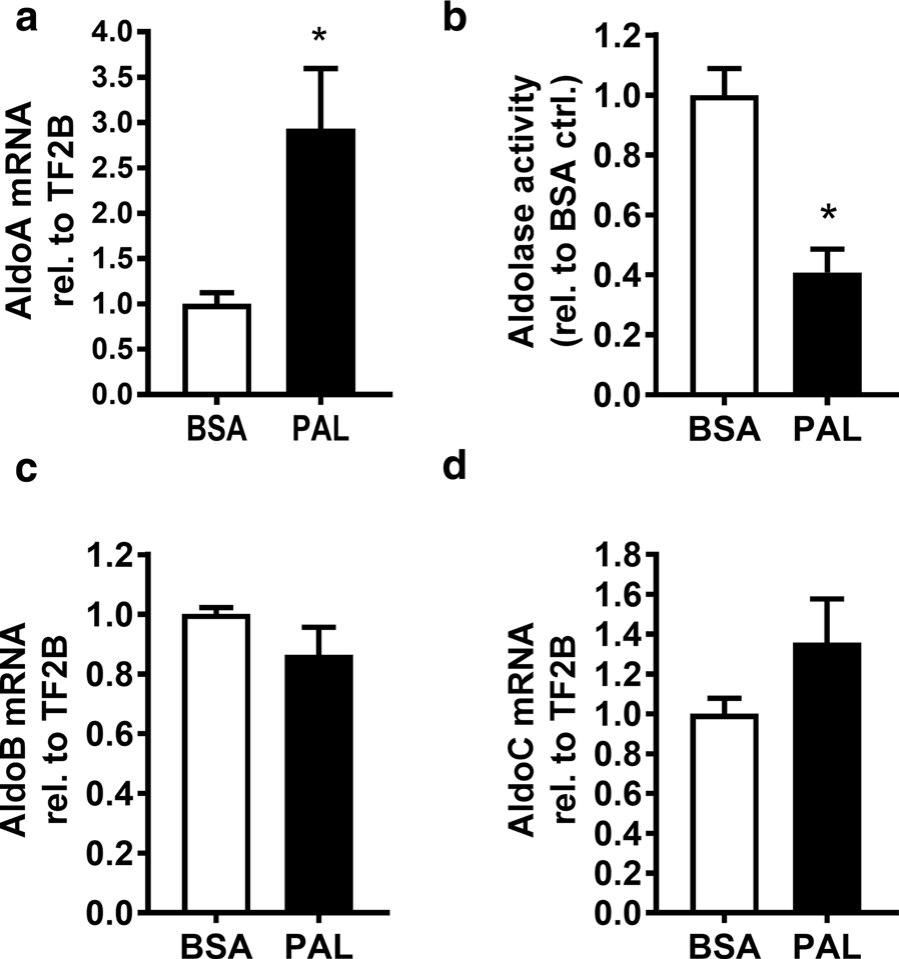
Enzyme activities and mRNA levels of aldolase of INS-1E cells treated with palmitate for 24 h. INS-1E cells were treated with palmitate or BSA for 24 h. **a** Cellular lysates were prepared and used to determine enzymatic activity of aldolase (Aldo). Raw activities were corrected for protein concentration. **b** Messenger RNA levels of *AldoA* was determined using RT-Q-PCR. Data are presented as mean fold change with controls set as 1, and are shown as mean ± SEM, N = 5–7 samples. *p < 0.05

A previously published mRNA array study (E-MTAB-3232 [13]) indicated that *AldoC* levels were increased threefold by palmitate treatment of another insulinoma cell line (INS-1 832/13 cells derived from the same parental cell line as the INS-1E subclone). To demonstrate utility of enzyme activity data, we analyzed activity and mRNA levels of aldolase enzymes in palmitate-treated INS-1E samples (Additional file 3, containing methods description for the case study). We found *AldoA* mRNA increased (2.9-fold of BSA control) after treatment (Fig. 2a), while enzymatic activity of aldo was decreased (0.5-fold of BSA control) (Fig. 2b). The *Aldo B* and *Aldo C* mRNA levels were unchanged by palmitate treatment (Fig. 2c–d). Although the underlying mechanisms remain elusive, these results demonstrate that supplementing RT-QPCR studies with enzyme activity data provides a valuable additional contribution for the biological interpretation of results.

### Discussion

In the present study, we used a sonication-based protocol for preparing lysates from six mammalian adherent cell lines followed by enzyme activity determination of *Aldo*, HK, G6PD, PGM, PGI and PFK (Fig. 1). The method is a considerable extension of an experimental procedure originally established for plant phenotyping [1, 2]. The advantage of the 96-well plate format is that it requires few cells (far less than one T25 culture flask) and only little hands-on time. With samples being run in triplicate and having one no-substrate negative control per sample, this enables 22 samples per plate. Thus, a single person can easily measure up to 200 samples per day, with the spectrophotometer read time being the limiting factor.

We could identify distinct fingerprints of the individual cell lines based on the activity signatures of the tested enzymes (Fig. 1). This likely reflects differences in the cell lines’ characteristics, such as degree of differentiation, activity of metabolism, and preference for metabolic pathways.

Furthermore, we tested the performance of the assays with regard to amount of cell extract (Fig. 1g, S1a–c), assay temperature (Fig. 1h, S1d–f), freeze–thaw sensitivity (Fig. 1i, S1g–i) and precision of the assays (Table 1). For these assays, initial data showed that 5 µL of cell extract of 1000 µL generated gave consistent results. Further, the data show that for the four extensively tested enzymes, a fivefold diluted extract yielded stable measurable enzyme activities, which makes it possible to scale-down cell culture samples and, thus, limit expenses, cell culture labour, and include scarce material into studies.

For dependency on temperature, we observed changed PGI and PGM activities at different temperatures of assays (S1e, f). This may be due to changed enzyme stability, degradation, or catalytic activity. However, all four assays performed well at both 25 °C and 30 °C. Thus, it is possible to work at ambient temperatures, but without temperature control greater inter-assay variability should be expected.

Altogether, the results indicate that the original procedure [1] is not limited to plant samples, but is also highly suitable for application with cell culture samples of mammalian cell lines.

## Limitations

The experimental setup was limited by several factors:

- Exclusive use of adherent cell lines (with focus on INS-1E cells).
- A low sample number per cell line (3–4 samples per passage and three passages were tested).
- Assay temperature was below the physiologically relevant 37 °C and could be further optimized.

## Supplementary information

**Additional file 1.** Enzyme activity assay excel template: excel file for enzyme activity assay setup support. The file contains a blank layout sheet for test sample and control sample distribution on a 96-well plate. Additional sheets contain master mix volume calculations for activity assays for Aldolase, Glucose-6-phosphate Dehydrogenase, Hexokinase, Phosphofructokinase, Phosphoglucose isomerase, and Phosphoglucosemutase. Adjustment of the desired number of control and test samples (grey fields) results in recalculation of required volumes. All sheets are printable on A4 paper and can be filled out digitally or manually. https://doi.org/10.6084/m9.figshare.7859801.

**Additional file 2: Figure S1.** Additional activity data of glucose 6 phosphate dehydrogenase (G6PD), phosphoglucose isomerase (PGI) and phosphoglucomutase (PGM) measured in INS-1E cells. **a**–**c** Dilution series of cell extracts (N = 3). Diluted samples were normalized to the level of the undiluted sample (set to 1). Data are shown as mean ± SEM. *, p < 0.05 for undiluted vs fivefold diluted, **, p < 0.01 for undiluted vs 20-fold diluted. **d**–**f** Temperature sensitivity was tested by assay performance at 25 °C and 30 °C. N = 4 cell extracts tested per temperature. Data are shown as mean ± SEM. *, p < 0.05, ***, p < 0.0001. **g**–**i** Sensitivity to freeze–thaw cycles was assessed using cell extracts that underwent 1 (I), 2 (II) or 3 (III) freeze–thaw cycles. N = 4 cell extracts tested per condition. Data are shown as mean ± SEM. **, p < 0.01, ***, p < 0.0001. https://doi.org/10.6084/m9.figshare.7859804.

**Additional file 3.** Case study on the effect of palmitate treatment on Aldolase activity. Additional methods used in the case study. The file describes experimental procedures of in vitro palmitate treatment and analysis of enzyme gene expression. Obtained data were aligned with results from enzyme activity assays. https://doi.org/10.6084/m9.figshare.7859777.

## Data Availability

The datasets used and/or analyzed during the current study are available from the corresponding author on request.

## Abbreviations

Aldo: aldolase
BSA: bovine serum albumin
G6PD: glucose-6-phosphate dehydrogenase
GPI: glucose-6-phosphate isomerase
GCK: glucokinase
HK: hexokinase
Pal: palmitic acid
PGI: phosphoglucoisomerase
PGM: phosphoglucomutase
PFK: phosphofructokinase
