# Supplementary figures and images for: Physiological phenotyping of mammalian cell lines by enzymatic activity fingerprinting of key carbohydrate metabolic enzymes: a pilot and feasibility study
Source: BMC Res Notes. 2019 Oct 22;12:682. doi: 10.1186/s13104-019-4697-y (PMC6805439; doi:10.1186/s13104-019-4697-y)

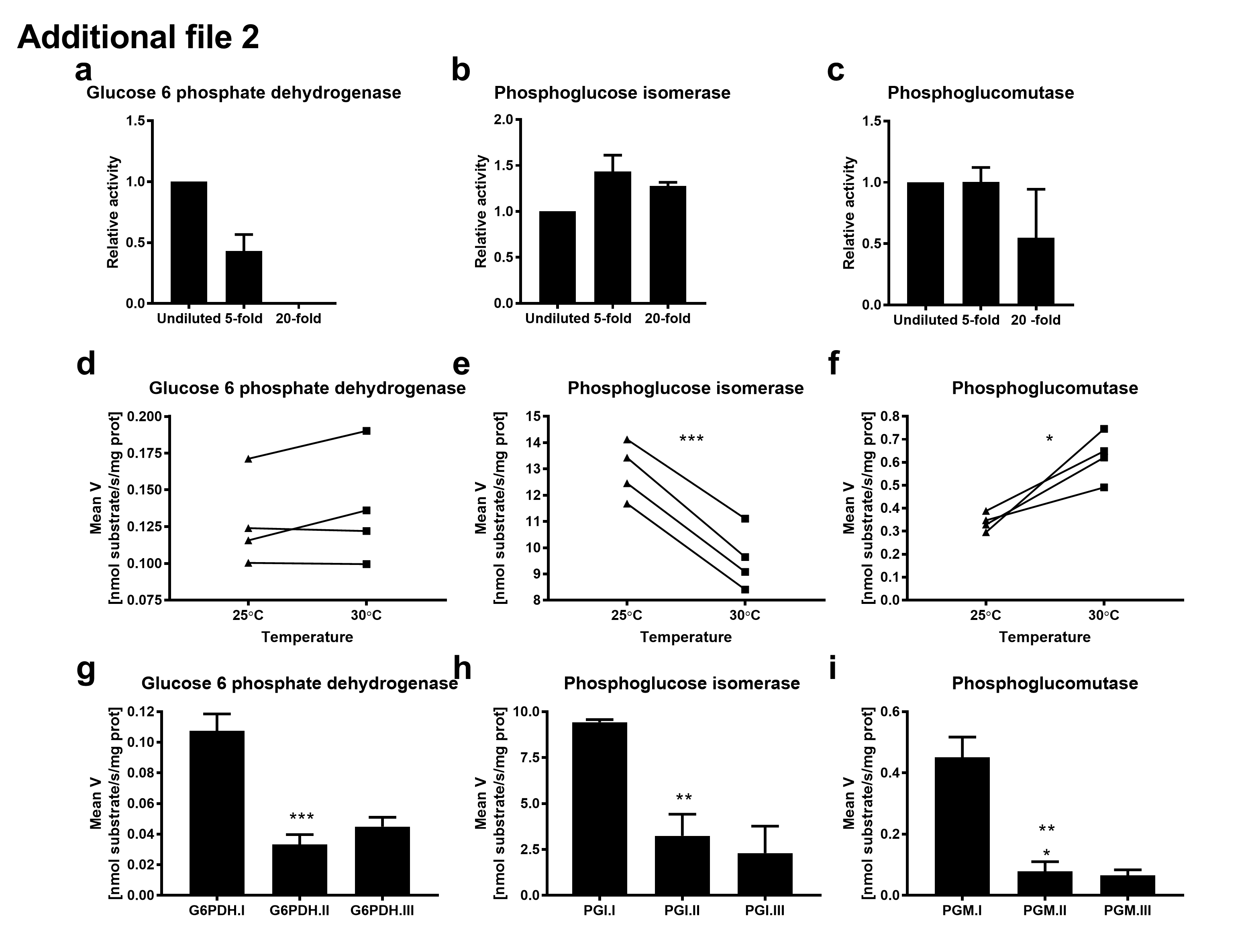

Supplement: Supplementary file 2 — Additional file 2: Figure S1. Additional activity data of glucose 6 phosphate dehydrogenase (G6PD), phosphoglucose isomerase (PGI) and phosphoglucomutase (PGM) measured in INS-1E cells. a–c Dilution series of cell extracts (N = 3). Diluted samples were normalized to the level of the undiluted sample (set to 1). Data are shown as mean ± SEM. *, p < 0.05 for undiluted vs fivefold diluted, **, p < 0.01 for undiluted vs 20-fold diluted. d–f Temperature sensitivity was tested by assay performance at 25 °C and 30 °C. N = 4 cell extracts tested per temperature. Data are shown as mean ± SEM. *, p < 0.05, ***, p < 0.0001. g–i Sensitivity to freeze–thaw cycles was assessed using cell extracts that underwent 1 (I), 2 (II) or 3 (III) freeze–thaw cycles. N = 4 cell extracts tested per condition. Data are shown as mean ± SEM. **, p < 0.01, ***, p < 0.0001. https://doi.org/10.6084/m9.figshare.7859804. [file 13104_2019_4697_MOESM2_ESM.tif]
