# Supplementary material for: Physiological phenotyping of mammalian cell lines by enzymatic activity fingerprinting of key carbohydrate metabolic enzymes: a pilot and feasibility study
Source: BMC Res Notes. 2019 Oct 22;12:682. doi: 10.1186/s13104-019-4697-y (PMC6805439; doi:10.1186/s13104-019-4697-y)
Supplement: Supplementary file 3 — Additional file 3. Case study on the effect of palmitate treatment on Aldolase activity. Additional methods used in the case study. The file describes experimental procedures of in vitro palmitate treatment and analysis of enzyme gene expression. Obtained data were aligned with results from enzyme activity assays. https://doi.org/10.6084/m9.figshare.7859777. [file 13104_2019_4697_MOESM3_ESM.docx]

**Additional file 3: Palmitate-induced alterations in enzyme gene expression and activity follow dissimilar trends in INS-1E β-cells**

A commonly used model for lipid-induced β-cell dysfunction, lipotoxicity, entails treatment of primary or clonal β-cells, such as INS-1E cells, with palmitate coupled to BSA. To investigate the action of lipotoxicity on INS-1E cells on the glucose metabolic enzyme fingerprint, we treated INS-1E cells according to a published lipotoxicity protocol for 24hrs before cell harvest. In parallel, we extracted RNA samples for comparison to mRNA levels.

**Methods**

***Palmitate treatment***

For the model of lipotoxicity in INS-1E cells palmitate was dissolved as a 200mM stock solution in ethanol (100%). For BSA-coupling, palmitate stock was diluted to 6mM concentration with BSA (10%, weight/volume) and shaken over night at 37°C. This corresponds to a molecular palmitate:BSA ratio of 4:1.The coupled palmitate was used for cell culture stimulation. For fatty acid treatment 48h after seeding, cells were stimulated for 24h with medium containing 0.25mM palmitate or BSA as control, as described. For the model of glucose toxicity in INS-1E cells; 24h after seeding medium was removed, cells washed with PBS and stimulated for 48h with medium containing either 5mM or 20mM glucose.

***RNA extraction and RT-Q-PCR***

For RNA isolation, medium was removed, and cells were harvested in 1mL TRI Reagent (SigmaAldrich). RNA extraction was performed as instructed by the manufacturer. RNA-concentration was determined using a Nanodrop ND-1000 spectrophotometer. Complementary DNA was synthesized from 1000 ng RNA using the SuperScript III First-Strand Synthesis kit (ThermoFisher) according to the manufacturer’s instructions using random hexamers. Q-PCR reactions were carried out with the Agilent MxPro3005 instrument using Quantitect SYBR green (Qiagen) according to manufacturer’s instructions. TFIIB was used as reference gene as it is unchanged by glucose and fatty acid treatment [1, 2]. Sequences (5’ to 3’) of used primers are AldoA (forward: GGCTTCTTTCACTGCACCAC, reverse: CCAGCTCCTTCTTCTGCTCC), AldoB (forward: CCGCTTGCAGGAACAAACAA, reverse: GTCCGAGATCCTCAGCACAG), AldoC (forward: GCTTGGACTGAGCTACTGTCTG, reverse: GGTATGAGTGGGGCATGGT), TFIIB (forward: gttctgctccaacctttgcct, reverse: tgtgtagctgccatctgcactt).

***Array data***

An mRNA array data from INS-1 cells treated with palmitate was obtained from Malmgren et al (2013)[3] ([E-MTAB-3232](https://www.ebi.ac.uk/arrayexpress/experiments/E-MTAB-3232/?query=malmgren)).

**References**

1. Ravnskjaer K, Boergesen M, Dalgaard LT, Mandrup S. Glucose-induced repression of PPARalpha gene expression in pancreatic beta-cells involves PP2A activation and AMPK inactivation. J Mol Endocrinol. 2006;36:289–99.

2. Dalgaard L, Thams P, Gaarn L, Jensen J, Lee Y, Nielsen J. Suppression of FAT/CD36 mRNA by human growth hormone in pancreatic β-cells. Biochem Biophys Res Commun. 2011;410:345–350.

3. Malmgren S, Spégel P, Danielsson APH, Nagorny CL, Andersson LE, Nitert MD, et al. Coordinate changes in histone modifications, mRNA levels, and metabolite profiles in clonal INS-1 832/13 beta-cells accompany functional adaptations to lipotoxicity. J Biol Chem. 2013;288:11973–11987.
